# Update of the FANTOM web resource: high resolution transcriptome of diverse cell types in mammals

**DOI:** 10.1093/nar/gkw995

**Published:** 2016-10-27

**Authors:** Marina Lizio, Jayson Harshbarger, Imad Abugessaisa, Shuei Noguchi, Atsushi Kondo, Jessica Severin, Chris Mungall, David Arenillas, Anthony Mathelier, Yulia A. Medvedeva, Andreas Lennartsson, Finn Drabløs, Jordan A. Ramilowski, Owen Rackham, Julian Gough, Robin Andersson, Albin Sandelin, Hans Ienasescu, Hiromasa Ono, Hidemasa Bono, Yoshihide Hayashizaki, Piero Carninci, Alistair R.R. Forrest, Takeya Kasukawa, Hideya Kawaji

**Affiliations:** 1 Division of Genomic Technologies (DGT), RIKEN Center for Life Science Technologie, 1-7-22 Suehiro-cho, Tsurumi-ku, Yokohama, Kanagawa 230-0045, Japan; 2 Genomics Division, Lawrence Berkeley National Laboratory, 84R01, 1 Cyclotron Road, Berkeley, CA 94720, USA; 3 Centre for Molecular Medicine and Therapeutics at BC Children's Hospital Research, Department of Medical Genetics, University of British Columbia, 950 West 28th Avenue, Vancouver, BC, V5Z 4H4, Canada; 4 Centre for Molecular Medicine Norway (NCMM), Nordic EMBL Partnership, University of Oslo, 0318 Oslo, Norway; 5 Department of Cancer Genetics, Institute for Cancer Research, Oslo University Hospital, 0372 Oslo, Norway; 6 Institute of Bioengineering, Research Center of Biotechnology, Russian Academy of Science, Leninsky prospect, 33, build. 2, 119071 Moscow, Russia; 7 Vavilov Institute of General Genetics, Russian Academy of Science, Gubkina str. 3, Moscow 119991, Russia; 8 Department of Biosciences and Nutrition, Karolinska Institutet, HÃ¤lsovÃ¤gen 7–9, 14183 Huddinge, Sweden; 9 Department of Cancer Research and Molecular Medicine, Norwegian University of Science and Technology (NTNU), P.O. Box 8905, NO-7491 Trondheim, Norway; 10 Program in Cardiovascular and Metabolic Disorders, Duke's National University of Singapore Medical School, 8 College Road, Singapore 169857, Singapore; 11 Department of Computer Science, University of Bristol, Merchant Venturers Building, Woodland Road, Bristol BS8 1UB UK; 12 The Bioinformatics Centre, Section for Computational and RNA Biology, Department of Biology, University of Copenhagen, Ole Maaloes Vej 5, DK-2200 Copenhagen, Denmark; 13 Section for Computational and RNA Biology, Department of Biology & Biotech Research and Innovation Centre, University of Copenhagen, Ole Maaloes Vej 5, DK-2200 Copenhagen, Denmark; 14 Database Center for Life Science (DBCLS), Joint Support-Center for Data Science Research, Research Organization of Information and Systems (ROIS), 1111 Yata, Mishima 411-8540, Japan; 15 RIKEN Preventive Medicine and Diagnosis Innovation Program, 2-1 Hirosawa, Wako, Saitama 351-0198, Japan; 16 Preventive medicine and applied genomics unit, RIKEN Advanced Center for Computing and Communication, 1-7-22 Suehiro-cho, Tsurumi-ku, Yokohama, Kanagawa 230-0045, Japan; 17 Systems biology and Genomics, Harry Perkins Institute of MedicalResearch, PO Box 7214, 6 Verdun Street, Nedlands, Perth, Western Australia 6008, Australia

## Abstract

Upon the first publication of the fifth iteration of the Functional Annotation of Mammalian Genomes collaborative project, FANTOM5, we gathered a series of primary data and database systems into the FANTOM web resource (http://fantom.gsc.riken.jp) to facilitate researchers to explore transcriptional regulation and cellular states. In the course of the collaboration, primary data and analysis results have been expanded, and functionalities of the database systems enhanced. We believe that our data and web systems are invaluable resources, and we think the scientific community will benefit for this recent update to deepen their understanding of mammalian cellular organization. We introduce the contents of FANTOM5 here, report recent updates in the web resource and provide future perspectives.

## INTRODUCTION

Recent advances in transcriptomics have improved the coverage as well as the detection accuracy of profiled RNA molecules. This means that several new opportunities are available for studying molecular function, gene regulation, embryogenesis, response to environmental stimuli and more.

The FANTOM project, one of the longest-lived collaborative projects in genomics, aims at the functional characterization of mammalian genomes. It started in the early 2000 with the generation of more than 100 000 mouse full-length cDNAs that revealed that the portion of the genome encoding for proteins is small, whereas the majority of it is involved in producing non-coding RNAs (1,2). Those full-length cDNAs were produced within both FANTOM1 and FANTOM2. FANTOM3 employed Cap Analysis of Gene Expression (CAGE) paired with first generation sequencing, allowing precise identification of genes transcriptional start sites (TSSs). The project uncovered the first comprehensive promoter landscape of a mammalian genome (3) and the existence of anti-sense transcription (4). FANTOM4 adopted CAGE and 454 Life Science sequencing combined to Illumina microarrays to study a model of differentiation in human THP-1 (myeloid leukemia) cells, and to define the transcriptional regulatory network based on TSS activity that explained such timely process (5). Several databases were developed to collect the results from those four FANTOM iterations: the FANTOM-db (6) to store the mouse cDNA clones; the RIKEN Expression Array Database (READ) (7) containing the expression profile data for the clones; and the FANTOM4 web resources (8) to integrates CAGE expression profiles with short RNA sequencing data and microarray data. The FANTOM4 web resource also incorporates genome browsers and bioinformatics analysis results.

Within the FANTOM5 project, the consortium profiled nearly 2000 human and 1000 mouse samples, representative of the majority of cell types and tissues, using CAGE followed by next generation single molecule sequencing (HeliScope) (9). FANTOM5 was organized in two phases; the first one focused on steady cellular states and the second was directed toward understanding transcriptional regulation changes upon differentiation, stimulation or infection. The main results of FANTOM5 were the most comprehensive promoter and enhancer atlases to date that could be generated using the same technology and the same platform (10,11). Not only are their mapped genomic coordinates provided, but also accurate activity profiles of promoters across samples and their association to genes, enhancers and cell ontology information can be obtained.

All the primary and processed data were packed together with genome browsers and network viewers (12) and are provided via a unique entry point (http://fantom.gsc.riken.jp) as the FANTOM web resource for easy access and navigation. In this paper, we introduce the contents of the resource and describe our updates following the initial release of the FANTOM5 web resource.

## RESOURCES FOR THE FIRST PHASE OF FANTOM5

The FANTOM web resource combines visualization tools and data archives, which are publicly accessible on the Internet (Table 2). All data described in our previous publication (12) were generated on the samples covering steady cellular states (573 and 128 primary cell samples from human and mouse, 152 human and 271 mouse tissues, and 250 human cell lines). Each sample was annotated with terms from the FANTOM5 ontology, which incorporates cell types, anatomical tissues and systems, as well as diseases, from ontologies in the Open Biomedical Ontologies (OBO) Library, including CL, Uberon and DO (13).

### Data archives

Data archives include basic processing outputs of the CAGE reads, such as genome alignments and 1-base resolution frequency of the alignment 5′-ends (corresponding to raw read counts of TSS activities monitored by CAGE). Additional analysis results, such as coordinates of CAGE peaks (corresponding to TSSs), their activities for the entire samples collection, their association with genes, transcribed enhancers, transcription factors and DNA motifs are also included.

### Data interfaces

In addition to the data files available for download, subsets of the data, such as expression tables of specific genes in a few samples, can be obtained by using the Table Extraction Tool (TET). Moreover, a BioMart interface (14) is available for CAGE peak annotation and RDF (Resource Description Format) query is supported in the form of nanopublications (15) Both tools help researchers with little computational skills to easily retrieve information from multiple databases.

The data are also accessible in a genome-centric manner in ZENBU (16) and via the Track Data Hub (17). The former allows dynamic visualizations of expression profiles together with basic data processing on the fly in a specified region of the genome. The latter, which is available as one of the public hub entries to the UCSC Genome Browser database (18), allows to visualize the data along with other public data resources such as ENCODE (19). Access to the FANTOM5 data hub can be faster when using the recently configured Asian mirror (genome-asia.ucsc.edu).

A network-oriented interface is provided by using BioLayout Express 3D (20), which provides sample-sample and promoter–promoter relationships as interactive three-dimensional networks. Other views, including sample-, gene- and promoter-centric views, are provided through FANTOM5 SSTAR (21); for example, a single page dedicated to each sample provides related information and analysis results, such as cell classification and highly expressed transcription factors.

## NEW DATA FOR THE SECOND PHASE OF FANTOM5

### Dynamic states of cells

The second phase of FANTOM5 aimed at studying dynamic changes in the transcriptional landscape over time, complementing the collection of steady cellular states of the first phase. We collected 19 human and 14 mouse time series, covering development (mouse visual cortex and cerebellum), *in vitro* differentiation (iPS to neurons, ES cells to cardiomyocite, calcification), response to drugs (MCF7 cells response to HRG and EGF, macrophage response to LPS) and infection (rinderpest, influenza), which resulted in additional nearly 1000 human and 600 mouse samples. The complete sets of FANTOM5 human and mouse samples are listed in Supplementary Tables S1 and S2, respectively.

### Identification of additional promoters and enhancers

Given the increase in CAGE profiles number, we extended the list of promoters and transcribed enhancers. As a result, the total number of identified peaks (that correspond to a promoter) has increased by 10% in human and 30% in mouse to a total of ∼200 000 and 158 000, respectively. Although the samples profiled in the phase 2 make up roughly 50% of the entire FANTOM5 data collection, the number of distinct cell types that was added is small and as a result does not expand the set of identified human promoters to the same extent as the previous phase. Transcribed enhancers were also identified by using the added CAGE profiles based on bi-directionality of transcription initiation (10), resulting in additional 20 000 human regions, while mouse enhancers were identified all at once in the second phase (Table 1).

**Table 1.** Summary of the number of samples, promoters and enhancers for human and mouse

Archived data	Human		Mouse	
	Phase 1	Phase 1+2	Phase 1	Phase 1+2
Samples	975	1816	399	1018
CAGE peaks	184 827	201 802	116 277	158 966
Enhancers	43 011	65 423	NA	44 459

### Upgrade to the latest genome assemblies

All data were originally processed based on the reference genomes GRCh37 (hg19) for human and GRCm37 (mm9) for mouse. Thanks to the continued efforts to improve the reference genome sequences, GRCh38 (hg38) and GRCm38 (mm10) have recently become available as the new target of genome annotation in the scientific community (18). We therefore compiled a data set that consists of (i) TSS activities at a single base-pair resolution from re-alignment of the CAGE reads with the latest genome assemblies, representing the most accurate TSS profiles (ii) CAGE peaks consistent with those defined on the old genome assemblies by using liftOver, a tool for conversion of genomic coordinates across different assemblies (https://genome.ucsc.edu/cgi-bin/hgLiftOver), and (iii) expression tables based on (i) and (ii) with dedicated normalization, representing the most accurate expression profiles of consistent promoters between two genome builds. Gene-promoter associations were also redone in order to account for changes in the coordinates of (ii) after migration to the latest genome assemblies.

## NEW FEATURES, DATABASES AND TOOLS IN THE SECOND PHASE OF FANTOM5

### Incorporation of the latest data and time series navigation

**Figure 1.**
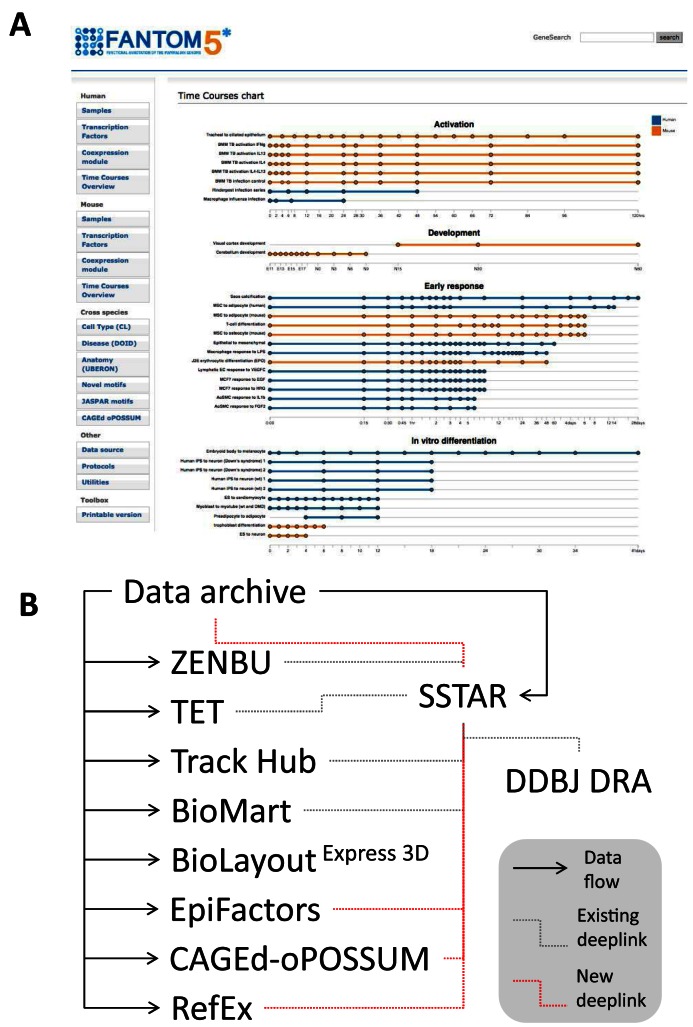
Recent update of SSTAR. (**A**) Clickable chart representing the set of time courses and their samples, (**B**) Hyperlinks from the SSTAR database.

Contents of all databases and interfaces (ZENBU, SSTAR, TET, Track Hub and BioMart) were expanded to cover the new data without the need to change their data structure. Besides the increase in data content, navigation interfaces to the 33 time series data sets were implemented in SSTAR, where a clickable chart representing the set of time courses (22) redirects users to individual time points or to a dedicated page for one set of time series (Figure 1A).

### Partner databases and tools

As a result of extensive use of the FANTOM5 data, multiple databases and tools were also developed by collaborating researchers to share their own results (Table 2). Most of them were published independently, but were hyperlinked with SSTAR at the content level (deep linking), as in Figure 1B. Here, we give a brief introduction of each, so to help users to explore contents and derived results, and developers to design their future studies based on the FANTOM data set.

**Table 2.** Lists of all databases and tools with access URLs

Database or tool name	Description	update from the initial release (Lizio *et al.*)	URL
data files	primary data archive	updated to include phase 2 data	fantom.gsc.riken.jp/5/datafiles/latest/
TET	Table Extraction Tool	updated to include phase 2 data	fantom.gsc.riken.jp/5/tet/
BioMart	database system for flexible querying based on data-agnostic modeling	updated to include phase 2 data	fantom.gsc.riken.jp/5/biomart/
nanopublication	the smallest unit of publishable information (nanopublication) for FANTOM5	none	antom5.nanopub.org/sparql
ZENBU	collaborative, omics data integration and interactive visualization system	updated to include phase 2 data	fantom.gsc.riken.jp/zenbu/
Track Hub	web-accessible directories of genomic data that can be viewed on the UCSC Genome Browser	updated to include phase 2 data	fantom.gsc.riken.jp/5/datahub/
BioLayout Express 3D	tool for the visualization and analysis of network graphs	none	fantom.gsc.riken.jp/5/biolayout/
SSTAR	database system to explore samples, transcriptional initiations, and regulators analyzed in the FANTOM5 project	updated to include phase 2 data	fantom.gsc.riken.jp/5/sstar/
CAGEd-oPOSSUM	motif enrichment analysis from CAGE-derived TSSs	added in phase 2	cagedop.cmmt.ubc.ca/CAGEd_oPOSSUM/
EpiFactors	database for epigenetic factors, corresponding genes and products	added in phase 2	epifactors.autosome.ru/
Ligand Receptor Connectome	visual guide to FANTOM5 Ligand-Receptor interactions	added in phase 2	fantom.gsc.riken.jp/5/suppl/Ramilowski_et_al_2015/
Mogrify	directory of defined factors for direct cell reprogramming	added in phase 2	www.mogrify.net/
SlideBase	Selection of cell or tissue specific genomic elements using sliders.	added in phase 2	slidebase.binf.ku.dk
RefEx	Data set of mammalian gene expression measured by different technologies	added in phase 2	refex.dbcls.jp

CAGEd-oPOSSUM (23) implements TF binding site (TFBS) enrichment predictions within *cis*-regulatory regions derived from TSSs identified by CAGE to infer key transcriptional regulators. The user selects TSSs associated with their cell or tissue type of interest and the tool predicts TFBSs within putative *cis*-regulatory regions surrounding the TSSs to assess their over-representations as compared to what would be expected by chance. CAGEd-oPOSSUM has been applied to all phase 1 FANTOM5 samples using TF binding motifs from the JASPAR database (24). By combining motif enrichment analysis with CAGE-derived *cis*-regulatory regions, CAGEd-oPOSSUM helps power insight into the TFs that act as key regulators in specific mammalian cells and tissues.

EpiFactors (25) is a manually curated database providing information about epigenetic regulators, their complexes, targets and products in human. It contains information on 815 proteins, including 95 histones and protamines and 69 complexes. For 789 of the related genes, expressions values across all FANTOM5 samples are presented. The protein (gene) page contains a summary of the available data with external links, including mouse orthologous from MGI (26) if available, and the complex page provides information on proteins involved in complex formation, their molecular function and specific targets and products.

Ligand Receptor Connectome (27) is a web-based visual and interactive guide to cell-cell communication networks between 144 major human primary cells (profiled in FANTOM5) using 2442 ligand–receptor pairs. Users can select their cells, ligands, receptors or interacting pairs of interest and visualize them as a network with cells being nodes and interactions being edges. This helps to uncover which cells are communicating the most via selected ligand-receptor pair(s), shows the top paths used to communicate between any given two cells or yields information on most specific ligands and receptors expressed by a given cell. Visualized networks can be downloaded as an *svg* image or in a format compatible with Cytoscape (28) for further exploration.

Mogrify (29) is a tool that can predict TFs that may be used for the reprogramming of cells by taking advantage of regulatory network information and gene expression data. The pre-calculated results, key regulators influencing the change of cellular states are based on the FANTOM5 data and are available in the database.

SlideBase is a web-based tool that enables users to select enhancers, promoters and more from the FANTOM project upon user-defined expression thresholds for each sample, through the usage of interactive sliders. This allows for on-the-fly selection of tissue-specific enhancers or promoters, with definitions set by the user. The tool also reports overlaps with SNPs, enhancer-TSS associations by co-expression and allows for genome-browser visualizations of selected sets.

RefEx (Reference Expression Data set) is a curated reference data set of mammalian gene expression measured by four different but complementary technologies (EST, GeneChip, CAGE and RNA-seq) from publicly available data. The FANTOM5 expression atlas for human and mouse was used for gene expressions in 40 tissues commonly used in RefEx, as well as cell lines, primary cells, adult and fetal tissues. They are visible in choropleth maps on 3D human body images from BodyParts3D (30) in addition to comparative histogram of gene expression levels.

## FUTURE PLANS

### Additional data

The published data so far have described samples derived from human and mouse. In the course of the FANTOM5 project, we attempted to achieve cross-species comparisons in a few selected cell types. Studies on rat, dog, chicken and macaque samples are under preparation for publication and will be incorporated to the FANTOM web resource.

A current limitation of the published data in FANTOM5, besides the coverage of species, lies in the approaches we take to explore RNAs. Since CAGE protocol is designed to capture only the 5′-end of capped long RNA molecules, the internal structure of long RNAs and small regulatory RNAs remains unexplored. To complement the CAGE profiles, CAGEscan (31), RNA-seq and small RNA sequencing data are being analyzed and will also be added to the FANTOM web resource.

### Additional databases and tools

As introduced above, the data set provided by FANTOM5 forms a foundation for unique analysis and tool development. We foresee efforts in the development of additional databases and interfaces, within and outside of the FANTOM consortium, and won't exclude the possibility to interconnect external tools with our databases; this would increase both their and the FANTOM web resource overall utility.

### Upgrade of the existing databases and interfaces

We are also actively working on upgrading the existing databases and interfaces. In particular, functionalities of ZENBU are being enhanced to empower users with more data manipulation and visualization tools. The backend engine of SSTAR, Semantic MediaWiki, is going to be upgraded to the latest version to improve responsiveness. These changes, as well as expansion of the contents to cover additional data, will further facilitate exploration and characterization of mammalian genomes in the context of cellular states.

Lastly, the consortium is already focused on the next FANTOM project. For its sixth iteration, we aim to uncover the function of long non-coding RNAs by high-throughput screening coupled with CAGE.
